# Primary Cilia and Their Role in Acquired Heart Disease

**DOI:** 10.3390/cells11060960

**Published:** 2022-03-11

**Authors:** Zachariah E. Hale, Junichi Sadoshima

**Affiliations:** 1 Department of Internal Medicine-Pediatrics, Rutgers New Jersey Medical School, Newark, NJ 07103, USA; zh203@njms.rutgers.edu; 2 Department of Cell Biology and Molecular Medicine, Cardiovascular Research Institute, Rutgers New Jersey Medical School, Newark, NJ 07103, USA

**Keywords:** primary cilia, congenital heart disease, ciliopathy, cardiomyopathy, heart failure

## Abstract

Primary cilia are non-motile plasma membrane extrusions that display a variety of receptors and mechanosensors. Loss of function results in ciliopathies, which have been strongly linked with congenital heart disease, as well as abnormal development and function of most organ systems. Adults with congenital heart disease have high rates of acquired heart failure, and usually die from a cardiac cause. Here we explore primary cilia’s role in acquired heart disease. Intraflagellar Transport 88 knockout results in reduced primary cilia, and knockout from cardiac endothelium produces myxomatous degeneration similar to mitral valve prolapse seen in adult humans. Induced primary cilia inactivation by other mechanisms also produces excess myocardial hypertrophy and altered scar architecture after ischemic injury, as well as hypertension due to a lack of vascular endothelial nitric oxide synthase activation and the resultant left ventricular dysfunction. Finally, primary cilia have cell-to-cell transmission capacity which, when blocked, leads to progressive left ventricular hypertrophy and heart failure, though this mechanism has not been fully established. Further research is still needed to understand primary cilia’s role in adult cardiac pathology, especially heart failure.

## 1. Introduction

The incidence of adults with congenital heart disease (CHD) has been progressively increasing for some time, in part driven by significant improvements in the management of these patients as children [1]. A child born with CHD today has a 97% chance of survival to adulthood [2], and, at least since 2010, the number of adults living with CHD has exceeded the number of children [3]. Further improvement will need to come from the ongoing management of these patients as adolescents and adults [2]. Adults with CHD show an increased risk of developing ventricular hypertrophy, heart failure, arrhythmias, and sudden cardiac death later in life than patients born with grossly normal hearts [4,5,6,7]. In fact, a majority of these patients die from cardiac causes [1].

Current strategies for the management of these patients, as well as for risk stratification, are insufficient [8,9]. In order to improve outcomes in these patients, providers and translational scientists need to understand the mechanisms of acquired heart disease in this population. With their strong links to both congenital and acquired heart disease, primary cilia represent an important target for further research and therapeutics.

Primary cilia have been the focus of research since the 1960s, when they were first recognized as distinct from motile cilia and present in most mammalian tissues [10,11]. Diseases related to cilia gene mutations, coined ciliopathies, have since been identified in many organ systems [12,13]. Cilia’s role in the cardiovascular system has been more recently defined, with large studies and reviews describing the occurrence of most, if not all, congenital heart diseases in response to mutations in cilia-related genes [14,15]. Primary cilia have now been recognized to play an important role in acquired heart disease as well, and the etiology of this association remains an active area of research. Here we review the available literature on primary cilia and their role in acquired heart disease, and outline areas where more research is needed.

## 2. Primary Cilia

### 2.1. Cilia Structure and Components

Primary cilia are extrusions of the plasma membrane that display a variety of receptors and mechanosensors. The core structure is an axoneme of nine doublet microtubules that extend from a basal body, and they are therefore referred to as “9 + 0” cilia. This distinguishes them from motile “9 + 2” cilia, which have an additional two dynein-associated central microtubules, permitting motion [11].

As primary cilia do not intrinsically have associated ribosomes, they instead rely on the intraflagellar transport (IFT) system to ferry receptors and other proteins into and out of the cilium [12]. This system is capable of bidirectional movement along the length of the flagella, between the outer doublet of microtubules and the flagellar membrane [16,17]. IFT proteins, especially Ift88, are often knockout targets in cilia research, as their inactivation results in the absence of primary cilia in the affected cell [18,19].

**Figure 1:**
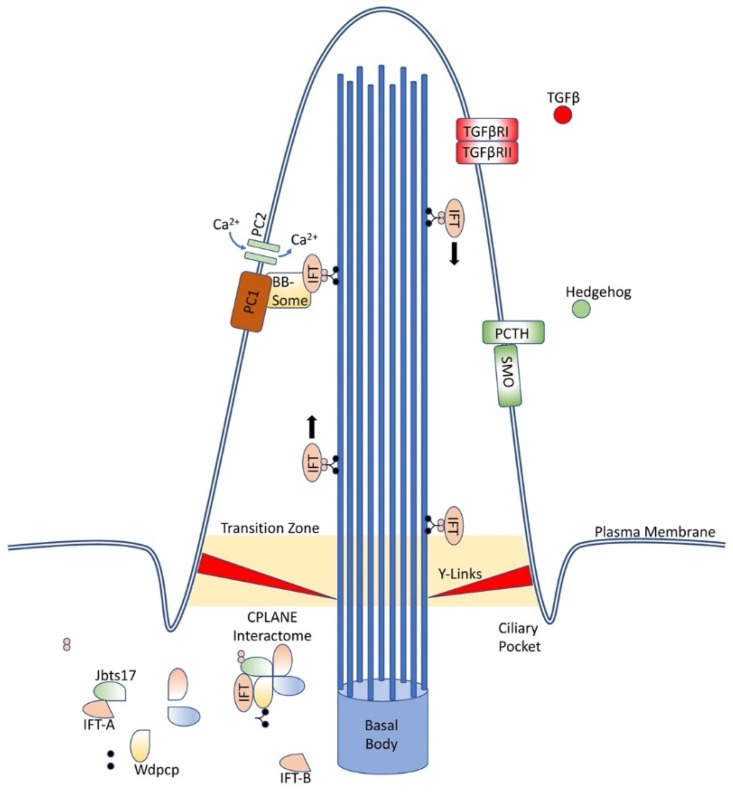
Primary cilia structure and components. Primary cilia are an extrusion from the cell wall capable of displaying numerous proteins, including those depicted here and many others, and are supported by nine doublet microtubules arising from the basal body. IFT proteins ferry components along the length of the cilia, while the CPLANE interactome remains at the base of the cilia.

At the base of the cilium, near the basal body, an interactome of proteins, coined CPLANE, is responsible for ciliogenesis and intraflagellar transport. (Figure 1) These proteins act at the basal body to recruit IFT-A proteins to the base of the cilium and stabilize and insert complete IFT-A particles into the axoneme. Mutations in these proteins have been associated with a variety of ciliopathies [17]. Numerous other membrane-bound proteins located along the cilia have been associated with ciliopathies as well, including polycystins, known for causing autosomal dominant polycystic kidney disease, and septins, which have been linked with a variety of cancers and neurodegenerative conditions [20,21,22,23,24,25].

### 2.2. Ciliopathies

For classification purposes, first-order ciliopathies are those diseases which occur due to a mutation in genes required for the proper assembly, maintenance, or function of the cilia or the related centriole; second-order ciliopathies occur due to dysregulation of further upstream factors, such as the nuclear transcription factors Atf3, Tsc22d4, and Cbx5 [26,27]. There are at least 300–1000 first-order, and many more second-order, genes [26,28,29].

Primary cilia play an important role in most mammalian organ systems, so ciliopathies tend to display a variety of multiorgan dysfunction phenotypes. (Table 1) Bardet—Biedl syndrome, for example, is characterized by retinitis pigmentosa, obesity, polydactyly, cognitive impairment, and renal failure [30]. Most ciliopathies show some amount of brain, craniofacial, or endocrine dysfunction, though kidney, reproductive, and heart tissues are also often involved [26].

One of primary cilia’s most important roles, and part of the reason mutations cause such varied phenotypes, is the display of receptors important for cell signaling pathways and the machinery for signal transduction [31]. One of the best studied is Hedgehog (Hh), which is highly dependent on functional primary cilia [19,32,33,34]. The transmembrane protein Smoothened (Smo), which is responsible for Gli protein activation in the Hh pathway, is found at the tip of the cilium [35]. Other pathways, such as Wnt, Notch, and PCP, similarly depend on primary cilia, and ciliopathies can impair their function [36,37].

Autophagy and programmed cell death pathways, which are important for tissue homeostasis and are perturbed in neurodegenerative diseases and cancer, depend on proper ciliary function due to machinery localization to the cilia and interdependent feedback mechanisms [36]. Loss of primary cilia function results in excess cell death from autophagy in mitochondrial stress responses and from mitochondria-dependent apoptosis [38,39]. Finally, extracellular matrix makeup is sensed and regulated through primary cilia [40,41].

### 2.3. Primary Cilia Locations

Despite their importance for many cellular pathways, primary cilia have not been identified on all cardiac cell types. Primary cilia are displayed on fibroblasts in the heart, Ref. [55] as well as on vascular endothelial cells, though expression on valvular endothelium decreases over time, from abundance in embryologic samples to near absence in adult samples [13,56,57]. Most cardiac interstitial (mesenchymal) cells also display primary cilia [57]. Cardiomyocytes contain primary cilia in embryonic tissue samples and lack them in adult samples, but there is disagreement regarding their presence on neonatal samples, Refs. [55,58] suggesting a possible loss of primary cilia over time.

## 3. Primary Cilia in Acquired Heart Disease

### 3.1. Acquired Valvular Heart Disease

The importance of proper cilia function in the embryonic heart has been well established [14,19,59,60]. In a comprehensive analysis of over 87,000 mutagenized mouse fetuses, Li et al. identified 61 genes in which mutations were capable of producing echocardiographically identifiable congenital heart defects, and 35 of these genes encoded either motile or primary cilia proteins. An additional 16 genes were involved in cilia-transduced cell signaling, and 10 regulated vesicular trafficking, which is necessary for proper cilia function [14].

Unlike the congenital defects analyzed by Li et al., mitral valve prolapse (MVP) is not evident on echocardiogram at birth. Instead, it is unusual in infants and children and it is more frequently identified in patients aged 30–80 years of age [61]. This valve pathology is a result of myxomatous degeneration over the lifetime of the patient.

In a genome-wide association study, enrichment for cilia genes was found in patients with MVP, and murine homozygous mutants of the two known familial MVP genes, *Dchs1* and *Flna*, showed decreased primary cilia length on the neonatal mitral valve leaflets [57,62]. Exploring cilia’s mechanistic role in MVP, Toomer et al. showed that the presence of primary cilia on endocardial cells correlated with increased proteoglycan and decreased collagen in the extracellular matrix of valve endocardium. Conditional knockout of intraflagellar transport protein 88 (Ift88) in cardiac endothelial cells in mice resulted in decreased primary cilia counts, increased proteoglycans, and fragmented collagen, i.e., the initiation of myxomatous degeneration [57,63]. While primary cilia abundance on valvular endothelium decreases with age, their effect on the extracellular matrix persists. As adults, these mice show myxomatous mitral valve disease [57].

### 3.2. Fibrosis

In addition to myxomatous degeneration of the valve, patients with MVP also show progressive left ventricular fibrosis. Cardiac fibrosis is an excessive production and deposition of scar tissue, often a result of conditions such as hypertension or diabetes mellitus, and can lead to increased tissue stiffness, cardiomyocyte atrophy, and arrhythmias [64,65]. The fibrosis observed with MVP is more significant than that seen in patients with primary mitral valve regurgitation from a non-MVP etiology, which may suggest a common cause for both excessive fibrosis and MVP [66].

In cardiac fibroblasts, activation of the transforming growth factor β-1 (TGF-β1) receptor results in production of fibronectin, collagen type I, and collagen type III, which are necessary components of the extracellular matrix in fibrotic tissue [67]. Fibroblasts also undergo transformation to myofibroblasts, which express α-smooth muscle actin (α-SMA) and display contractile ability.

Inactivation of primary cilia by small interference RNA (siRNA) silencing of Polycystin-1 (PC1) in fibroblasts results in a lack of upregulated collagen production in response to TGF-β1. Similarly, siRNA silencing of either PC1 or Ift88 in cardiac fibroblasts results in failure of the fibroblasts to differentiate into myofibroblasts capable of contractile function, which is necessary for standard cardiac remodeling. These mice instead show excess myocardial hypertrophy and altered scar architecture [55].

In addition to native cardiac fibroblast proliferation, endothelial-mesenchymal transition (EndMT) is now recognized as an important source of fibroblasts for perivascular and subendocardial fibrosis [68]. Knockdown of Ift88 in endothelial cells, which results in the absence of primary cilia on these cells, appears to be insufficient to directly induce EndMT in vivo but may prime these cells for EndMT in response to lower stress than would otherwise be required [69,70].

### 3.3. Vascular Pathology and Cilia

In addition to their role in fibrosis after an ischemic injury, primary cilia also regulate atherosclerosis and, therefore, the risk of ischemic events. Primary cilia serve as mechanosensors in a variety of cell types [71]. In endothelial cells with functional primary cilia, excess shear stress stimulates PC1 interaction with Polycystin-2 (PC2), permitting calcium influx and activating calcium-dependent signaling molecules, including calmodulin and calcium-dependent protein kinase (PKC), that lead to activation of endothelial nitric oxide synthetase (eNOS) and subsequent vasodilation [72,73,74].

Branch points and the lesser curvature of the aorta are at particular risk of atherosclerosis due to relatively low and oscillatory shear stress [75]. These areas also display increased density and stability of primary cilia [76,77]. Initial research suggested that primary cilia may play a role in producing atherosclerosis, as apolipoprotein-E-deficient (*Apoe^−/−^*) mice display increased primary cilia as well as increased atherosclerosis at these risk points [63]. However, removing these cilia via knockout of Ift88 results in increased atherosclerosis in *Apoe^−/−^* mice in response to a high fat, high cholesterol diet, suggesting this is a protective response mediated by eNOS [78].

PC1 and PC2 gene mutations produce autosomal dominant polycystic kidney disease (ADPKD), which results in hypertension in two-thirds of cases [79]. In addition to the eNOS activation mechanism, primary cilia also protect against hypertension via dopamine receptor 5 (DR5) [74,80]. Stimulation at this receptor results in adenylyl cyclase and PKC activation, leading to vasodilation [81].

### 3.4. Ventricular Remodeling and Recovery

Cardiomyocyte hypertrophy is an important cell autonomous and non-cell autonomous adaptive response to significant stress, especially hypertension, that is necessary for survival. However prolonged stress and resultant excess hypertrophy and cardiac remodeling can lead to heart failure and sudden cardiac death [82,83,84]. Cardiomyocytes have some ability to sense mechanical forces, including hemodynamic stress, in order to convert stress into intracellular growth signals and induce hypertrophy. However, the molecular identity of the mechanosensor remains elusive. Primary cilia are an attractive candidate as a mechanosensor; however, this has not been demonstrated experimentally.

One possible mechanism appears to be via ciliary extracellular-like vesicles (cELVs) [85]. These vesicles are released from cilia under normal circumstances and at increased rates under fluid shear stress. Blocking ciliary proteins necessary for cELV production using short hairpin RNA (shRNA) prevents cELV production and results in left ventricular hypertrophy, decreasing left ventricular ejection fraction, and, eventually, low blood pressure and cardiovascular collapse [85,86].

### 3.5. Congenital Heart Disease and Late-Onset Heart Failure

Patients with CHD show a higher risk of heart failure later in life than patients born with grossly normal hearts [4,5,6]. One study showed an overall prevalence of heart failure of 26% in a cohort of patients with surgically corrected CHD [6]. While the highest risk of heart failure is in patients with morphologically right ventricles exposed to systemic pressures, even patients with isolated ventricular septal defect are at higher risk of systolic and diastolic dysfunction 30 or more years after surgical repair [87]. This suggests that either a factor of the surgery can produce ventricular dysfunction decades later, such as the residual scar tissue, or else that a common etiology for both the CHD and ventricular dysfunction exists.

Some familial CHD-producing gene mutations have also been associated with ventricular dysfunction, such as the sarcomeric gene *MYH7* [5]. However, we are not aware of any current published research directly linking primary cilia gene mutations with heart failure through a mechanism different than those discussed above. While primary cilia are not displayed on adult cardiomyocytes, many ciliary proteins continue to exist and function at non-cilia locations, and cilia continue to be present in other cell types. Acquired ventricular dysfunction may therefore be mediated by ciliated non-myocytes, or else via cilia-independent functions of cilia proteins in cardiomyocytes. Alternatively, ciliogenesis may be reactivated in de-differentiated cardiomyocytes or cardiomyocyte progenitor cells in response to stress. Another possibility is that primary cilia defects in the developing heart result in permanent differences in the adult myocytes’ response to the stresses discussed above. Additional research is needed to identify the role of primary cilia in heart failure.

## 4. Concluding Remarks

Primary ciliary gene defects have previously been observed in a variety of syndromes, including ADPKD and Bardet—Biedl, as well as isolated congenital heart diseases. The role of cilia in these congenital conditions has been well defined. However, the role of primary cilia in acquired heart disease has not previously been reviewed.

Here we have reviewed literature exploring the effect of cilia gene knockouts on a variety of acquired cardiac pathologies. Mice with Ift88 knockout in valvular cells show myxomatous degeneration of the mitral valve similar to that observed in adult humans with mitral valve prolapse. Similarly, knockout in endothelial cells increased rates of endothelial to mesenchymal transition and increased fibrotic disease in response to stress. These models also show increased hypertension and atherosclerotic disease. Finally, primary cilia have cell-to-cell transmission capacity which, when blocked, leads to progressive left ventricular hypertrophy and heart failure.

While primary cilia have been linked with conditions that lead to heart failure, such as hypertension or atherosclerotic disease, a mechanistic causal relationship has not yet been fully established. Further research is needed to understand primary cilia’s role in adult cardiac pathology and especially in ventricular dysfunction.

Overall, despite decreased abundance in adult heart tissue, primary cilia continue to play an important role in cardiac homeostasis throughout adult life.

## Figures and Tables

**Table 1:** Known human ciliopathies. A list of known human ciliopathies, though many more are thought to be related to cilia function. Ciliopathies present with significant variation in phenotype depending on the underlying gene mutation and other factors. Due to historical clinical definitions, some syndromes are phenotypes possible from a variety of gene mutations, while others are phenotypic variations of the same genetic defect [42,43].

Ciliopathy Syndrome	Associated Genes
Alström syndrome [44]	ALMS1
Bardet—Biedl syndrome [30]	BBS1-16
Ellis-van Creveld syndrome [45]	EVC/EVC1, EVC2
Jeune syndrome (Asphyxiating thoracic dystrophy) [46]	IFT80
Joubert syndrome [47]	CEP290, others
Leber Congenital Amaurosis [48]	GUCY2D, RPE65, others
McKusick—Kaufman syndrome [49]	MKKS/BBS6
Meckel—Gruber syndrome [50]	MKS1-13, others
Nephronophthisis [51]	NPHP1-NPHP11, others
Orofaciodigital syndrome 1 [42]	OFD1
Polycystic Kidney Disease [21]	PKD1, PKD2
Senior—Løken syndromes [52]	NPHP1, NPHP3, others
Sensenbrenner syndrome (Cranioectodermal dysplasia) [53]	IFT122, WDR35
Short-rib polydactyly syndrome [54]	DYNC2H1
